# Maturing of public-private-people partnership (4P): Lessons from 4P for triple disaster and subsequently COVID-19 pandemic in Fukushima

**DOI:** 10.7189/jogh.12.03028

**Published:** 2022-07-08

**Authors:** Yurie Kobashi, Yuzo Shimazu, Yuki Sonoda, Hiroaki Saito, Makoto Yoshida, Masaharu Tsubokura

**Affiliations:** 1 Department of Radiation Health Management, Fukushima Medical University School of Medicine, Fukushima City, Fukushima, Japan; 2 Department of General Internal Medicine, Hirata Central Hospital, Hirata, Ishikawa district, Fukushima, Japan; 3 Department of Nursing, Jyoban Hospital, Tokiwa Foundation, Fukushima, Japan; 4 Department of Gastroenterology, Sendai Kousei Hospital, Miyagi, Japan; 5 Teikyo University School of Medicine, Itabashi-ku, Tokyo, Japan

Public-Private-People Partnership (4P) is a cross-sector collaboration combining “people” with a traditional Public-Private Partnership (PPP). The term “people” encompasses civil society organisations, academia, professional organisations, media, and others. 4P was proposed to compensate for the shortcomings in conventional PPPs regarding a lack of residents' viewpoints, clarification of civil engagement, and incorporation of bottom-up strategies, especially in disaster management [1,2]. People – the 4th P – can provide the missing connection in traditional PPPs to strengthen the partnership further and achieve effective and integrated partnerships between multiple sectors [3].

Cross-sectoral collaborations such as PPPs are very significant in the context of disaster medicine [4] since recovery from disasters requires the involvement of multiple sectors of a community [5]. Disasters are inherently local events and municipalities in affected areas carry the greatest responsibility in dealing with them, making cross-sector partnerships in such areas vital [6]. Further, recovery through cross-sector collaboration can increase a community’s capacity and capability [6]. Besides, local disaster management can be divided into the phases of “responding”, “recovering”, and “building resilience” [7]. Discussions on how 4P experiences across these longitudinal phases in the local community can help in dealing with subsequent crisis disasters can be useful; however, there are few reports on how knowledge and experience of local cross-sector collaborations have helped in disaster response and recovery.

The Fukushima prefecture experienced a triple disaster – the radiation disasters, the Fukushima Daiichi Nuclear Power Plant accident, and the Great East Japan earthquake. Cross-sector collaboration between the local government, private and public medical sectors, and the community has been ongoing for over 10 years in this disaster-affected area [8,9]. In fact, 4P among the local government, private sector, academic sector, professional institutions, and media has been carried out to promote civil-focused reconstruction. The areas recovering from the triple disaster have also been affected by the COVID-19 pandemic. The COVID-19 pandemic is considered a disaster with capacity enhancement being one of the major challenges for disaster damage management. Here, 4P is considered an essential solution [6]. This area is suitable for reporting how experiences and knowledge of cross-sector collaboration regarding disaster response and recovery can help with preparing for unexpected disasters in communities.

**Figure:**
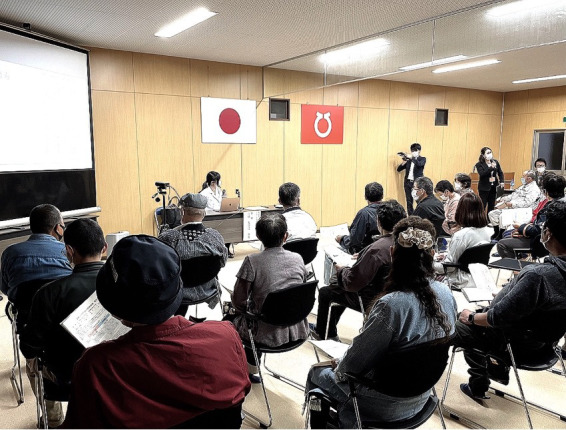
Photo: Doctors explaining the COVID-19 antibody testing results during a meeting with residents at Hirata Village governmental office as part of the Public-Private-People Partnership (4P). (The authors’ own collection, used with permission).

**Figure 1:**
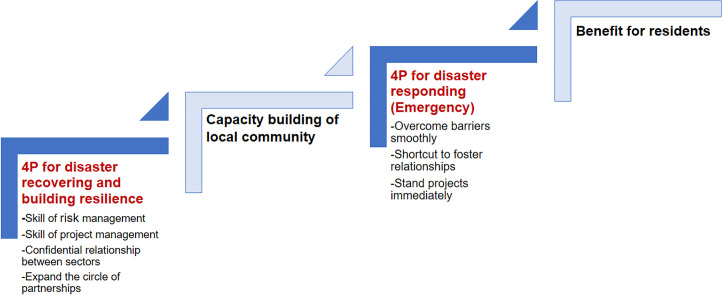
Maturating stages of Public-Private-People Partnership (4P). When the community experienced a disaster, the community was required to deal with the issues, and the skills and relationship between sectors might be mature. When the next disaster happens, the community would overcome the barriers and make strategy smoothly.

The COVID-19 pandemic has also affected the Fukushima prefecture, where post-disaster recovery is ongoing. In this area, the Fukushima Vaccination Community Survey (FVCS) – a series of antibody testing against COVID-19 – is carried out under multi-sectoral collaboration between research agencies, universities, local governments, and the medical sector. Approximately 2500 residents in the radiation disaster-affected areas, where the population is aging due to radiation, are participating in the project. Antibody testing among medical personnel, patients, frontline workers, and the general public is being carried out every three months. Fukushima prefecture might be one of the fastest cohort setting areas for COVID-19 antibody testing in the Japanese community. There are many barriers to cross-sector collaboration during disasters, including economic, political, legal, structural, procedural, strategic, executive, human, socio-cultural, and sharing of information [4,6]. Nevertheless, FVCS could overcome these barriers immediately. This paper presents the experience and knowledge of disaster recovery in the region and how it helped in responding to the newly occurring disaster and developing countermeasures, including the FVCS, for COVID-19 under 4P (**Figure 1**).

The first key point is that some municipalities in Fukushima prefecture already have many years of experience with surveys on radiation exposure and related health issues in the disaster-affected area. Therefore, these municipalities could deal with ethical matters in research without any hurdle. The persons involved in FVCS have a shared sense of value that makes invisible health threats visible, since sharing the results with the residents is vital. This helped to overcome several barriers – legal, structural, procedural, human, and socio-cultural. The accumulated experience of joint research, conferences, and workshops for radiation health management with the collaboration of municipalities, universities, hospitals, and residents has clarified the aspects that might give rise to ethical issues. The experience of 4P with radiation disaster has been valuable in overcoming such issues effectively in the COVID-19 pandemic era.

The second key point is that the FVCS principal investigator has built trust over the years with key persons in the sectors. Presently, a better balance could be maintained by working across municipalities, which would help the project overcome political and human barriers. Three municipalities cooperated with the FVCS, with the key persons from each municipality being the mayor (also the director of the medical institution), the head of the health and welfare department in a municipal organisation, the hospital director, the hospital office manager, the municipal hospital’s clinical director, and the head of a local medical association. Strong connections with the few key persons in the sectors who could make decisions and coordinate were essential. In this case, personal connections worked well instead of contracts and mere appearances.

The third key point was to expand cooperation to various sectors (based on key persons) such as hospitals (public and private) and health facilities, local government, several universities, media, local medical associations, non-profit organisations, research agencies, and others. COVID-19 has reduced profit in some hospitals; however, the economic, executive, and legal costs were met outside the budget obtained from the COVID-19 research agency. Notably, in a depopulated and aging rural community where the FVCS was carried out, collaboration with multiple sectors was essential, as medical facilities were limited by unmet needs. Medical institutions have the advantage of medical resources, while local governments have the advantage of access to the individual population. Therefore, it was important for the project that cooperation with each sector be achieved to compensate for weaknesses and overcome barriers.

The disaster-affected area of Fukushima prefecture, which experienced triple disasters, has dealt quickly with the unexpected disaster of COVID-19, with the residents benefiting from projects such as FVCS and the fastest COVID-19 vaccination drive in the prefecture. This has been possible thanks to more than a decade of radiation disaster recovery experience with 4P. Sustained efforts were effective in improving the quality of 4P. In other words, regular cross-sectoral collaboration and 4P development is necessary for effective countermeasures during emergencies and preparing for unexpected disasters in the long term.
